# Transcriptional silencing of ETS-1 abrogates epithelial-mesenchymal transition resulting in reduced motility of pancreatic cancer cells

**DOI:** 10.3892/or.2014.3613

**Published:** 2014-11-21

**Authors:** CHUNYAN LI, ZHONGHAN WANG, YAN CHEN, MIN ZHOU, HAIJUN ZHANG, RONG CHEN, FANGFANG SHI, CAILIAN WANG, ZONGDAO RUI

**Affiliations:** 1 Department of Oncology, Zhongda Hospital, Medical School of Southeast University, Medical School of Southeast University, Nanjing, Jiangsu 210009, P.R. China; 2 Department of Internal Medicine, Nanjing Government Hospital, Medical School of Southeast University, Nanjing, Jiangsu 210009, P.R. China; 3 Department of General Surgery, Zhongda Hospital, Medical School of Southeast University, Nanjing, Jiangsu 210009, P.R. China

**Keywords:** ETS-1, cell motility, epithelial-mesenchymal transition, pancreatic cancer cells

## Abstract

v-ets erythroblastosis virus E26 oncogene homolog 1 (ETS-1) plays crucial roles in a spectrum of malignancies. ETS-1 has gained attention in cancer research for its importance in cell migration, invasion and proliferation. In the present study, we focused on the effect of ETS-1 on epithelial-mesenchymal transition (EMT), which is characterized by reduced E-cadherin expression and increased N-cadherin expression. We found that *ETS-1* mRNA expression was positively correlated with *N-cadherin* and negatively correlated with *E-cadherin* mRNA expression in five pancreatic cancer cell lines. To elucidate the functionality of ETS-1 on EMT in pancreatic cancer cells, we constructed a green fluorescent protein (GFP)-expressing plasmid carrying ETS-1 short hairpin RNA (shRNA), and transfected Panc-1 cells with the plasmid. We detected reduced N-cadherin and vascular endothelial growth factor yet higher E-cadherin expression in the ETS-1-silenced cells compared with the control group. In addition, we observed reduced cell migration and increased adhesion in these cells. Our data showed that ETS-1 actively functioned as a regulator of EMT in Panc-1 cells, and provide additional evidence supporting a fundamental role for ETS-1 in metastatic pancreatic cancer cells. These results suggest that analysis of ETS-1 expression levels may provide an avenue for evaluating prognosis in pancreatic cancer.

## Introduction

Pancreatic cancer is the fourth leading cause of cancer-related mortality with a median overall 5-year survival of 5% worldwide (1). Patients at advanced stages face a dire median overall survival of less than one year (2). The lethal nature of pancreatic cancer is marked by its high potential for metastasis to the lymphatic system and distant organs (3), which prompted us to investigate the mechanisms involved in motility and metastasis.

v-ets erythroblastosis virus E26 oncogene homolog 1 (ETS-1) is the founding member of the ETS oncogene family, members of which possess a characteristic DNA-binding domain (ETS domain) of 85 amino acids (4,5). The ETS-1 protein controls the expression level of a multitude of genes including other transcription factors, proteases, cell cycle regulation genes, apoptosis-related genes, cytokines and growth factors (6). Upregulated ETS-1 has been observed in breast cancer, lung cancer, ovarian cancer, colorectal cancer and malignant melanoma (7–11). ETS-1 is overexpressed in invasive breast cancer and is correlated with the poor prognosis of breast cancer patients (12,13). High expression of ETS-1 promotes cell migration, invasion and anchorage-independent growth, while low ETS-1 expression reduces adherence of HeLa cells (14). One study showed that fibronectin-stimulated cell adhesion and migration of glioma U251 cells were suppressed by the expression of a dominant-negative form of ETS-1 (15). These studies demonstrated that ETS-1 plays major roles in the migration and invasion of cancer cells. Notably, ETS-1 is barely detectable in normal human pancreatic tissue, but high levels of expression are found in samples from human pancreatic cancer biopsies (16). Thus, there is compelling evidence showing that ETS-1 is involved in the migration and invasion of pancreatic cancer cells; however, the mechanisms by which ETS-1 mediates these effects have not been fully elucidated.

Epithelial-mesenchymal transition (EMT) facilitates malignant tumor progression and metastatic spread by enabling cancer cells to depart from the primary tumor, invade surrounding tissue and disseminate to distant organs (17). EMT is characterized by reduced E-cadherin expression and increased N-cadherin expression (17–19). E-cadherin is an adhesion molecule of epithelial cells, whose expression is frequently downregulated in invasive cancers. N-cadherin is associated with higher invasive potential, and is typically expressed by mesenchymal cells (20). It has been shown that expression of E-cadherin is increased in stably ETS-1-overexpressing cells, indicating that ETS-1 by itself has no activity to induce EMT in human squamous carcinoma cells (21). However, the results indicate that ETS-1 functions as one of the effectors of EMT (21). Additionally, EMT is usually accompanied by aberrant expression of vascular endothelial growth factor (VEGF) (22,23). Mukherjee *et al* (24) showed that high ETS-1 and VEGF expression is correlated with tumor angiogenesis, lymph node metastasis and poor patient survival in esophageal squamous cell carcinoma.

In the present study, we examined mRNA expression levels of *ETS-1*, *E-cadherin* and *N-cadherin* in five pancreatic cancer cell lines. We probed the influence of ETS-1 silencing on the expression of the EMT-related molecules, E-cadherin, N-cadherin and VEGF, and we investigated the effects of transcription factor ETS-1 on the motility of Panc-1 pancreatic cancer cells. Our data showed that ETS-1 functions as a regulator of EMT in pancreatic cancer cells, and suggest that analysis of ETS-1 expression levels may provide an avenue for evaluating prognosis in pancreatic cancer.

## Materials and methods

### Pancreatic cancer cell lines and cell culture

Human pancreatic cancer cell lines, Panc-1, PaTu-8988t, SW1990, Capan2 and BxPC3, were cultured under standard conditions (37°C in a humidified atmosphere with 5% CO_2_) in Dulbecco’s modified Eagle’s medium (DMEM) (Gibco, Carlsbad, CA, USA) supplemented with 0.1 mM non-essential amino acid, 10% fetal bovine serum (FBS) and 1% penicillin-streptomycin (all from Invitrogen, Carlsbad, CA, USA).

### Reverse transcription-polymerase chain reaction (RT-PCR)

Total RNA was extracted from stably transfected cells with TRIzol reagent (15596-026; Invitrogen), and first-strand cDNA was synthesized according to the manufacturer’s instructions (DRR036A; Takara, Tokyo, Japan). RT-PCR analysis was carried out using 2X Power Taq PCR Master Mix (PR1700; BioTeke, Beijing, China) under the following conditions: 95°C for 5 min to denature cDNA, followed by 30 cycles of 95°C for 30 sec, 57°C for 40 sec, and 72°C for 45 sec, followed by a terminal extension for 10 min at 72°C. The products were analyzed by electrophoresis on 2% agarose gels (16550-100; Invitrogen) at 120 V for 40 min, and the bands were visualized by an UltraPower™ Gel Imaging System (EP2018; BioTeke). The primer sequences are listed in Table I. All of the primers were obtained from Invitrogen.

### Short hairpin RNA transfection

Panc-1 cells (5×10^5^) were seeded in each well of a 6-well plate and were transfected with 500 ng/ml of either pSi-ETS1 (PIEE102075355) or control plasmids (both from Genechen, Shanghai, China) coupled with Lipofectamine 2000 (11668-019; Invitrogen). Following transfection, the culture medium was replaced by Opti-MEM medium (31985-062; Invitrogen) according to the manufacturer’s instructions.

### Real-time reverse transcription PCR (qRT-PCR)

Total RNA was extracted from stably transfected cells with the TRIzol reagent (15596-026; Invitrogen), and first-strand cDNA was synthesized according to the manufacturer’s instruction (DRR036A; Takara). qRT-PCR was carried out using LightCycler^®^ DNA Master SYBR-Green I as reaction mix (12015099001; Roche, Branchburg, NJ, USA) on the ABI 7300 Real-Time PCR system (Applied Biosystems, Foster City, CA, USA) under the following conditions: 95°C for 30 sec to denature cDNA and primers, followed by 40 cycles at 95°C for 5 sec, 60°C for 20 sec and 72°C for 30 sec, followed by a terminal extension for 7 min at 72°C. Gene expression was calculated with the comparative Ct method and normalized against the endogenous levels of glyceraldehyde-3-phosphate dehydrogenase (GAPDH). The primer sequences are listed in Table I.

### Western blot analysis

The transfected cells were washed with phosphate-buffered solution (PBS) and lysed with RIPA buffer containing a protease inhibitor cocktail (11873580001; Roche). The supernatant was collected after centrifuging the cell lysate (13,300 × g for 10 min), and the concentration of the cellular protein was measured using a BCA detection kit (P0010S; Beyotime, Jiangsu, China). The protein concentration was adjusted to 2 μg/μl for electrophoresis on a 10% SDS-PAGE gel. Cellular proteins separated on the gel were transferred to a polyvinylidene difluoride membrane (03010040001; Roche). After blocking with 5% nonfat milk in Tris-buffered saline containing 0.1% Tween-20, the membrane was incubated with specific primary antibodies: anti-GAPDH antibody (MB001, 1:5,000 diluted; Bioworld, St. Louis, MN, USA), anti-VEGF antibody (ab46154, 1:1,000 diluted), anti-ETS-1 antibody (ab26096, 1:1,000 diluted) (both from Abcam, Cambridge, UK), anti-N-cadherin antibody (BS2224, 1:1,000 diluted) and anti-E-cadherin antibody (BS1098, 1:1,000 diluted) (both from Bioworld). After incubation with the appropriate horseradish peroxidase-conjugated secondary antibody (LK-RAG007; Multi Sciences Biotech Co., Hangzhou, China), signals were detected using an enhanced chemiluminescence reagent (WBKLS0500; Millipore, Darmstadt, Germany) and subjected to the Alpha Innotech FluorChem FC2 Imaging system (Alpha Innotech Corp., San Leandro, CA, USA).

### In vitro cell migration assay

A scratch assay was utilized to examine *in vitro* cell migration of pancreatic cancer cells. After being transfected for 24 h, Panc-1 cells (5×10^5^ cells) were seeded in a 6-well plate and allowed to form a confluent cell monolayer, which was then scratched by a sterile pipette tip. The monolayer was washed with PBS, and fresh medium supplemented with 1% FBS (Invitrogen) was added. An inverted fluorescence microscope system (DMI3000B; Leica Microsystems, Heerbrugg, China) was used for imaging immediately after wounding (0 h) and after 12 h. The distances between the cell boundaries were measured using TPView (Shanghai Weitu Optics and Electron Technology Co., Ltd., Shanghai, China).

### In vitro cell adhesion assay

Cell adhesion assay was performed to quantify the ability of the cancer cells to adhere to fibronectin (F2006; Sigma-Aldrich, St. Louis, MO, USA). After 24 h of transfection, the ETS-1 short hairpin RNA (shRNA)-transfected and control cells were collected and seeded into a 96-well plate at 5×10^3^ cells/well and into a 12-well plate/well, which were both pre-coated with 20 μg/ml fibronectin for 30 min. Cells were allowed to adhere for 2 h at 37°C in a humidified atmosphere of 5% CO_2_. Unbound cells were removed by inverting and gentle washing in PBS. Cells in 96-well plates were stained with crystal violet solution containing 0.4 mg/ml crystal violet and 5% formaldehyde in PBS for 20 min at room temperature. The plate was then washed twice with de-ionized water and air-dried for scanning. The number of adherent cells at the bottom of each well of the 12-well plate was determined by a Countstar Automated cell counter (IC 1000; Inno-Alliance Biotech Inc., Wilmington, DE, USA).

### Statistical analysis

The Student’s t-test (two-tailed) was applied in the analysis of statistical significance. All data are presented as mean ± standard deviation. A P-value <0.05 was considered to indicate a statistically significant difference.

## Results

### Analysis of ETS-1, N-cadherin and E-cadherin mRNA expression in pancreatic cancer cell lines

**Figure 1:**
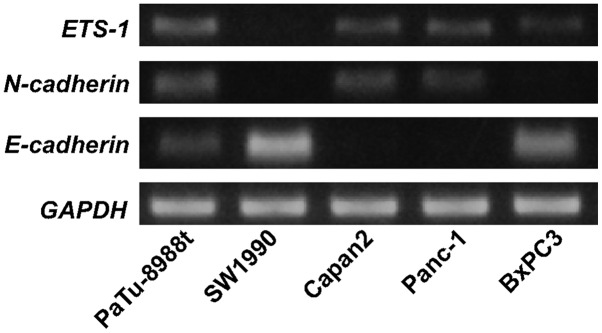
Analysis of mRNA expression levels of *ETS-1*, *N-cadherin* and *E-cadherin* in five pancreatic cancer cell lines. RNA levels of *N-cadherin*, *E-cadherin* and *ETS-1* were determined by RT-PCR in five pancreatic cancer cell lines. *GAPDH* was included as a normalization control. *ETS-1,* v-ets erythroblastosis virus E26 oncogene homolog 1; *GAPDH,* glyceraldehyde-3-phosphate dehydrogenase.

To evaluate the correlation between ETS-1 and EMT-related molecules, we analyzed the expression levels of *ETS-1*, *N-cadherin* and *E-cadherin* by RT-PCR in the following pancreatic cancer cell lines: PaTu-8988t, SW1990, Capan2, Panc-1 and BxPC3. The result showed that only SW1990 cells were negative for *ETS-1* mRNA expression. High *ETS-1* expression was positively associated with the expression levels of *N-cadherin* and negatively with the expression of *E-cadherin* in the PaTu-8988t, Capan2 and Panc-1 cells. In the SW1990 and BxPC3 cells, low *ETS-1* expression was correlated with high levels of *E-cadherin* but low levels of *N-cadherin* (Fig. 1). These results are consistent with a role for ETS-1 in EMT.

### Efficacy of the ETS-1 transcriptional silencing

**Figure 2:**
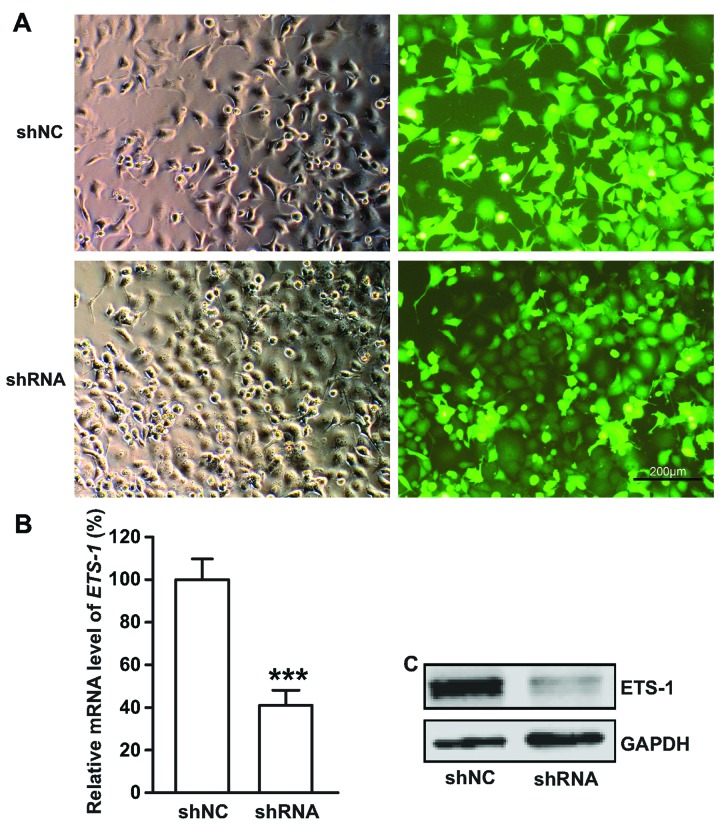
Efficacy of ETS-1 transcriptional silencing. Expression level of *ETS-1* in the Panc-1 cells was decreased after *ETS-1* shRNA transfection. (A) Brightfield and fluorescence images (magnification, ×100). (B) qRT-PCR analysis. Data are presented as mean ± SD of three independent experiments; ^***^P<0.001. (C) Lysates from *ETS-1* shRNA- and control shNC-transfected cells were subjected to western blot analysis; GAPDH was included as a normalization control. ETS-1*,* v-ets erythroblastosis virus E26 oncogene homolog 1; GAPDH, glyceraldehyde-3-phosphate dehydrogenase.

We performed ETS-1 inhibition in the Panc-1 cancer cell line, which exhibited relatively high expression of ETS-1 (Fig. 1). ETS-1 inhibition was performed using a green fluorescent protein (GFP)-expressing adenoviral vector carrying an shRNA targeting the *ETS-1* gene. The success of the plasmid transfection was monitored by green fluorescence (Fig. 2A). The silencing efficiency of the ETS-1 shRNA was determined using qRT-PCR and western blot analysis. qRT-PCR showed a 70% reduction in mRNA expression in the ETS-1 shRNA-transfected cells (Fig. 2B). The level of ETS-1 protein was also significantly decreased in the ETS-1-silenced cells (Fig. 2C).

### Transcriptional silencing of ETS-1 inhibits epithelial-mesenchymal transition

**Figure 3:**
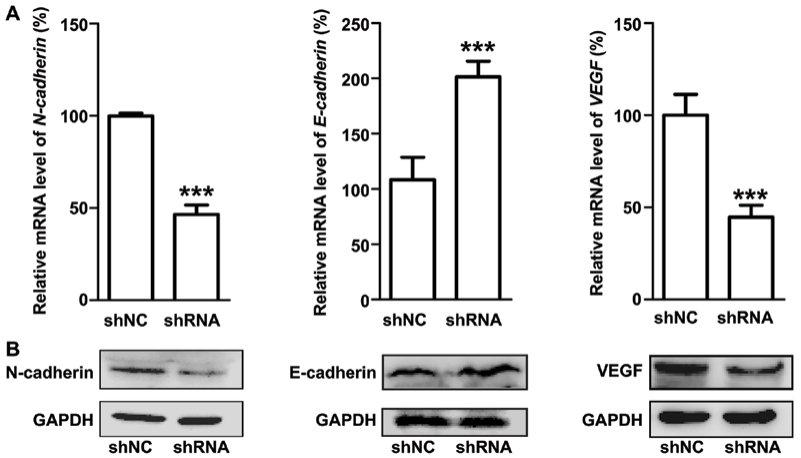
Effect of silencing of ETS-1 on expression of EMT-associated genes. (A) qRT-PCR analysis of *ETS-1*, *N-cadherin*, *E-cadherin* and *VEGF* mRNA expression in the transfected Panc-1 cells. *GAPDH* was included as a normalization control. (B) Western blot analysis of protein expression of corresponding molecules in the Panc-1 cells after ETS-1 silencing. ETS-1*,* v-ets erythroblastosis virus E26 oncogene homolog 1; EMT, epithelial-mesenchymal transition; *VEGF,* vascular endothelial growth factor; GAPDH, glyceraldehyde-3-phosphate dehydrogenase.

We investigated the effect of ETS-1 silencing on EMT by examining the expression levels of EMT-related proteins. In the ETS-1 shRNA-transfected cells, the level of *N-cadherin* mRNA expression was reduced to half, while the level of *E-cadherin* mRNA expression was increased as much as 2-fold (Fig. 3A). Consistently, western blot analysis revealed that ETS-1-shRNA plasmid transfection significantly decreased N-cadherin expression and increased E-cadherin expression in the Panc-1 cells (Fig. 3B). Our results also showed decreased expression of VEGF in the ETS-1 shRNA-transfected cells both at the mRNA (Fig. 3A) and protein expression levels (Fig. 3B).

### Transcriptional silencing of ETS-1 reduces cell migration, but increases cell adhesion

**Figure 4:**
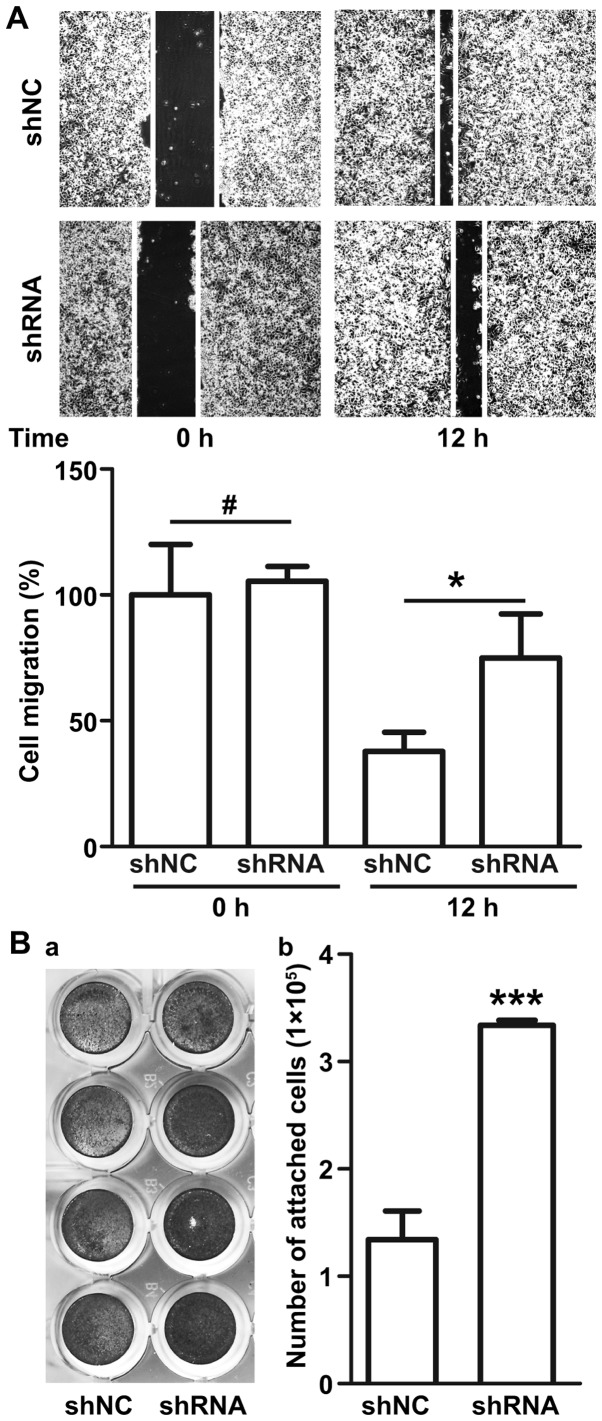
Reduced cell migration and increased cell adhesion in the Panc-1 cells after ETS-1 silencing. (A) ETS-1 silencing reduced the migration of Panc-1 cells. Phase contrast images of cultures were captured immediately after wounding (0 h) and after 12 h. Cell motility was quantified by measuring the distance between boundaries of the wounds. Data are presented as mean ± SD from three independent experiments; ^***^P<0.001. (B) ETS-1 silencing increased the attachment of Panc-1 cells. a, Crystal violet staining; b, adherent cell number. Data are presented as mean ± SD from three independent experiments; ^***^P<0.001. ETS-1*,* v-ets erythroblastosis virus E26 oncogene homolog 1.

Migration and invasion of cancer cells are key steps in tumor metastasis. The effect of transcriptional silencing of ETS-1 on the motility of Panc-1 cancer cells was measured in scratch and adhesion assays. The results of the scratch assay showed that ETS-1 silencing led to decreased cell migration. At 12 h, the distance between the scratch boundaries was 37% wider in the ETS-1 shRNA-transfected cells (Fig. 4A). Crystal violet staining assay showed that adhesion increased in the ETS-1 shRNA-infected cells (Fig. 4B-a). Following washing, an ~2.5-fold more ETS-1 shRNA transfected cells than control cells remained attached to the plate (Fig. 4B-b). These results showed that ETS-1 inhibition was highly effective in increasing the adhesion and reducing the motility of Panc-1 cells.

## Discussion

Patients with pancreatic cancer have an extremely poor prognosis due to the malignant behaviors of this disease. Invasion and metastasis are two main events associated with the poor prognosis. The prognosis of patients with clinically aggressive pancreatic cancer cannot be accurately predicted by standard variables such as pathologic stage; thus other biomarkers are needed. Expression of transcription factors associated with metastasis have shown promise as prognostic biomarkers (25,26). Little is known, however, regarding the expression of transcription factors involved in the processes regulating invasion and metastasis in pancreatic cancer. In the present study, we showed that the transcription factor ETS-1 plays important roles in regulating EMT and may have prognostic value.

EMT is characterized by downregulation of E-cadherin expression and acquisition of mesenchymal markers including N-cadherin, vimentin and fibronectin, which lead to loss of cell adhesion facilitating cell motility (27,28). Studies investigating the migration and invasion of malignant cancers have mostly associated ETS-1 with the expression of extracellular matrix metalloproteinase (13,21), integrins (29,30) and cadherins (27,31). One study showed that expression of c-ets-1 mRNA was associated with the EMT-derived phenotype typified by the expression of vimentin and the lack of E-cadherin in breast carcinoma cell lines (31). However, the influence of ETS-1 on the expression of N-cadherin and E-cadherin involved in the EMT-derived phenotype has not been investigated in pancreatic cancer cells. In this study, associations between expression of *ETS-1* and EMT-related molecules *N-cadherin* and *E-cadherin* were investigated. We found that ETS-1 mRNA was expressed in four of five pancreatic cancer cell lines. Furthermore, the *ETS-1* mRNA expression level was positively associated with the mRNA expression level of *N-cadherin* but was negatively associated with that of *E-cadherin*. To further evaluate the role of ETS-1 during EMT, we inhibited *ETS-1* expression with an ETS-1-specific shRNA and assessed its effects on the expression of N-cadherin, E-cadherin and VEGF in the Panc-1 cell line, a highly metastatic epithelial-pancreatic adenocarcinoma cell line. Our data showed that transcriptional silencing of ETS-1 induced a switch from N-cadherin to E-cadherin expression in Panc-1 cells, which indicates a block in EMT. In addition, our results showed that ETS-1 inhibition reduced VEGF expression. This is consistent with the previous finding that VEGF expression is significantly associated with ETS-1 expression (32). Hence, by demonstrating a close relationship between ETS-1 and EMT-related molecules, we provide strong evidence that ETS-1 plays a crucial role in the process of EMT in pancreatic cancer cells.

Aberrant expression of ETS-1 was found to be correlated with malignant cancer behaviors such as tumor proliferation, invasion, migration and angiogenesis (9,33,34). Our previous study also showed that gambogic acid-loaded magnetic Fe_3_O_4_ nanoparticles could inhibit proliferation and migration of Panc-1 cells via inactivating ETS-1 (35). Expression of ETS-1 has also been shown to decrease adhesion in endothelial (36), HeLa (14) and U251 glioma cells (15). However, the mechanisms of ETS-1 involvement in the EMT process have not been investigated in pancreatic cancer cells. Our results revealed that ETS-1 inhibition led to EMT downregulation. Cells undergoing EMT become invasive and migratory. Therefore, to determine whether ETS-1 silencing would reduce the motility of Panc-1 pancreatic cancer cells, we performed scratch and adhesion assays. We demonstrated, as shown in Fig. 4, that ETS-1 silencing reduced cell migration and increased cell adhesion. Our results suggest that ETS-1 promotes cell migration and invasion by promoting EMT.

In conclusion, our data support that ETS-1 plays functionally significant roles in the metastasis of pancreatic cancer cells by regulating the expression of N-cadherin and E-cadherin involved in epithelial-mesenchymal transition. We also found that ETS-1 silencing inhibits the expression of VEGF, which has been reported to be a probable marker for poor prognosis (37). Although its mechanism remains incompletely understood, our results suggest the potential utility of ETS-1 as an adverse prognostic factor, and highlight the need for further research to elucidate the role and importance of ETS-1 during the progression of pancreatic cancer.

## Figures and Tables

**Table I:** Sequences of the oligonucleotide primers.

Gene	Sequence (5′-3′)	Product (bp)
*ETS-1*	Forward: 5′-GTCGTGGTAAACTCGG-3′ Reverse: 5′-CAGCAGGAATGACAGG-3′	246
*N-cadherin*	Forward: 5′-AGTGAGCCTGCAGATTTTAAGGTGGATG-3′ Reverse: 5′-CACTTGCCACTTTTCCTGGGTCTCTT-3′	132
*E-cadherin*	Forward: 5′-TTGCACCGGTCGACAAAGGAC-3′ Reverse: 5′-TGGAGTCCCAGGCGTAGACCAA-3′	140
*VEGF*	Forward: 5′-AACCAGCAGAAAGAGGAAAGAGG-3′ Reverse: 5′-CCAAAAGCAGGTCACTCACTTTG-3′	133
*GAPDH*	Forward: 5′-CCACCCATGGCAAATTCCATGGCA-3′ Reverse: 5′-TCTAGACGGCAGGTCAGGTCCACC-3′	251

*ETS-1*, v-ets erythroblastosis virus E26 oncogene homolog 1; *VEGF*, vascular endothelial growth factor; *GAPDH*, glyceraldehyde-3-phosphate dehydrogenase.
